# The lived experience of food insecurity among adults with obesity: a quantitative and qualitative systematic review

**DOI:** 10.1093/pubmed/fdae016

**Published:** 2024-02-26

**Authors:** Rebecca Briggs, Hope Rowden, Lukasz Lagojda, Timothy Robbins, Harpal S Randeva

**Affiliations:** Warwick Medical School, University of Warwick, Coventry CV4 7AL, UK; Warwick Medical School, University of Warwick, Coventry CV4 7AL, UK; Warwickshire Institute for the Study of Diabetes, Endocrinology and Metabolism (WISDEM), University Hospitals Coventry and Warwickshire NHS Trust, Coventry CV2 2DX, UK; Clinical Evidence-Based Information Service (CEBIS), University Hospitals Coventry and Warwickshire NHS Trust, Coventry CV2 2DX, UK; Warwick Medical School, University of Warwick, Coventry CV4 7AL, UK; Warwickshire Institute for the Study of Diabetes, Endocrinology and Metabolism (WISDEM), University Hospitals Coventry and Warwickshire NHS Trust, Coventry CV2 2DX, UK; Warwick Medical School, University of Warwick, Coventry CV4 7AL, UK; Warwickshire Institute for the Study of Diabetes, Endocrinology and Metabolism (WISDEM), University Hospitals Coventry and Warwickshire NHS Trust, Coventry CV2 2DX, UK; Centre for Sport, Exercise and Life Sciences, Research Institute for Health & Wellbeing, Coventry University, Coventry CV1 5FB, UK; Institute of Cardiometabolic Medicine, University Hospitals Coventry and Warwickshire NHS Trust, Coventry CV2 2DX, UK

**Keywords:** food choice, food environment, obesity

## Abstract

**Background:**

Food insecurity and obesity are increasing both globally and in the UK. In this review we systematically assess the lived experiences of people with obesity who are food insecure and often turn to food banks.

**Methods:**

We systematically searched electronic databases from January 2007 until October 2022. Data from eligible studies were extracted and the studies assessed for quality. Thematic analysis and narrative synthesis approach was used to analyse the extracted data.

**Results:**

Six themes were identified among 25 included studies, including: the financial cost of food; psychological aspects related to food insecurity; geographical access and the food environment; food practices in the home; experience of food assistance; and parental-child relationships. The cost of healthy food and psychological factors were identified as key driving factors of the relationship between food insecurity and obesity. Psychological factors such as depression, low self-esteem and stress played an important part in the lived experience of people with obesity and food insecurity.

**Conclusion:**

The food environment provides context in which food decisions are made, therefore, systems change is necessary to ensure families can afford the food that enables a healthy diet. For clinicians, identification, and attention to the impact of food insecurity on people with obesity are important.

## Introduction

Food insecurity (FI) occurs as a result of insufficient economic, social and physical resources to access food or adequate nutritional value in order to meet one’s basic needs.^1^ Disruptions in food supply chains and the rising costs of energy, exaggerated by the COVID-19 pandemic and the war in Ukraine and extreme climate events, are factors contributing to food poverty and malnutrition globally and in the UK.^2^^,^^3^ This recent emergence of a global cost of living crisis and the resulting increase in FI pose a significant risk for escalating the obesity epidemic and associated conditions, including Type 2 diabetes, cardiovascular disease and poor mental health.^4–7^ Between April and September 2022, emergency food parcel provisions in the UK increased by 52% to >1.3 million compared to those in 2021.^8^ While recent systematic reviews found that food banks struggle to provide enough food to meet to meet nutritional guidance,^9^^,^^10^ the prevalence of obesity among food bank users remains higher compared to the general population.^11^

In 1992, Dietz observed a causal relationship between FI and obesity and hypothesized that coincidence of poor nutrition and increased weight may be related.^12^ The association is portrayed as paradoxical as FI is often associated with limited resources, whereas obesity is attributed to abundance.^13^ This ‘food insecurity-obesity paradox’ was first reviewed in 2007 by Dinour *et al*. after observing a positive association between FI and increased weight in women.^14^ Recently, multiple systematic reviews conducted in both high-income and low-middle-income countries found multiple positive relationships between the incidence of increased weight among individuals exposed to FI, although this association remains less clear in men.^5^^,^^15^^,^^16^ Importantly, a recent systematic review with meta-analysis found that adults who are food insecure are statistically more likely to have obesity.^10^

The ‘insurance hypothesis’ proposes that evolutionary mechanisms may increase the intake of food to accumulate energy stores for periods of time when food is scarce.^17^ For example, food assistance users (people who utilize food banks, food pantries or funding from the government to assist with the cost of food) who are unable to retain food stamps throughout each given month tend to develop a ‘feast and famine’ relationship with food.^6^^,^^14^

Furthermore, the low cost of ultra-palatable, calorie-dense but low in nutritional value, processed foods are common narratives throughout conversations about FI and obesity,^5^^,^^6^^,^^15^^,^^18^ further emphasizing the role of food assistance and eating habits.

There is a recurring theme within the literature examining the relationship between FI and obesity in which obesity is perceived to be a result poor decision-making,^19^^,^^20^ and it is consistent with the internalized stigma that depicts people with obesity as insatiable and lacking willpower.^21^ Furthermore, studies of attitudes towards food bank users found that there is a debate whether people with obesity should benefit from food assistance programmes.^1^ The so-called ‘fat shaming’ leads to negative psychological outcomes and self-destructive behaviours, including emotional and binge eating.^22^ Hence, it is important to consider the role of psychological factors in eating behaviours of people experiencing FI.

FI is increasingly a chronic problem rather than a temporary invonvenience.^9^ The ongoing cost of living crisis is expected to exacerbate this phenomenon, which emphasizes the need to consider a life-course perspective and the socioeconomic disadvantages experienced by people who are obese and food-insecure.^13^ The experiences of people with obesity and FI have not yet been systematically analysed. Therefore, the aim of this review is to summarize the current evidence to provide a better understanding of the lived experiences of people with obesity exposed to FI.

## Methodology

This systematic review was conducted according to the Preferred Reporting Items for Systematic reviews and Meta-Analyses (PRISMA) guidelines.^23^ The protocol for this systematic review was prospectively registered with the international prospective register of systematic reviews, available at https://www.crd.york.ac.uk/prospero/display_record.php?ID=CRD42022368762.

### Criteria for considering studies for inclusion

Inclusion criteria were created within the Population-Interest-Context (PICo) framework^24^ and are shown in Table 1. Studies were included if they fulfilled the following criteria: (i) had data available for adults (18+) with obesity (BMI ≥ 30 kg/m^2^) and (ii) the participants were food-insecure, including usage of foodbanks or stakeholder views on obesity and FI. Studies were excluded if extraction of data for only those participants with obesity and FI was not possible or mean BMI of participants was not in obese range. All methodologies were eligible for inclusion if inclusion and exclusion criteria were met.

**Table 1:** PICo framework for study inclusion

Population of interest	Adults (18+) with obesity
Interest	FI, food banks, food assistance programmes
Context	FI—obesity paradox

### Search methods

Bibliographic reference databases, including MEDLINE, Web of Science, EMBASE, Applied Social Sciences Index and Abstracts (ASSIA), Allied and Complementary Medicine (AMED) and APA PsychINFO were searched for peer-reviewed literature related to FI and obesity in the context of financial crisis from January 2007 until October 2022.^25^ Search strategies designed in consultation with an academic librarian (see Acknowledgements) used a combination of related terms and synonyms of the concepts: obesity, FI and cost of living (see Supplementary Table 1). Searches were limited to literature published in English. Reference lists of included studies were hand-searched for any additional studies and google searches were conducted for grey literature.

### Study selection

**Fig. 1:**
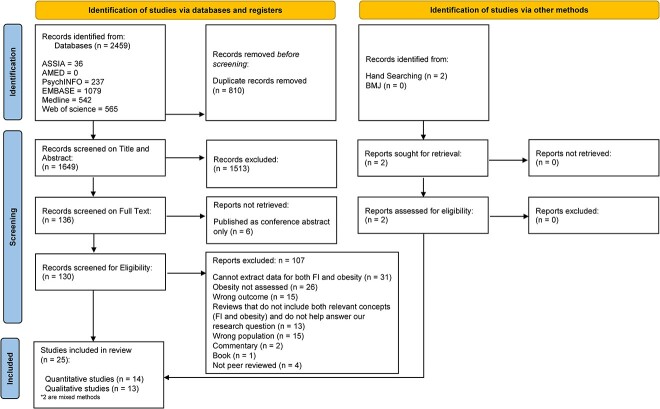
PRISMA diagram of study selection.

The study selection process is outlined in Fig. 1. The articles were imported into EndNote reference management software and duplicates identified. The de-duplicated citations were exported into Rayyan software for study selection.^26^ Two authors (RB and HR) conducted independent and blinded screening of abstracts and full text articles. Any disagreements at either the abstract or full text screening stage were resolved by a third reviewer (TR).

### Data extraction

A data extraction template was developed by two review authors (HR and RB) and piloted independently for suitability using a random sample of both qualitative and quantitative studies, and minor adjustments to the template were made following a discussion. Each reviewer extracted data (by alphabetical order) from half of the total number of included papers. Qualitative data was extracted into EPPI reviewer software.^27^

### Quality appraisal

Included studies were appraised using criteria outlined by Greenhalgh.^28^ Checklists were chosen for each article according to individual study design (see Supplementary Appendix 2 and 3).

### Data analysis

#### Thematic synthesis

Thematic synthesis was performed using the approach described by Thomas and Harden.^29^ Descriptive codes were created for themes identified in the literature. Line-by-line coding of papers was conducted using EPPI reviewer.^27^ Analytical themes representing the experiences of people with obesity and FI were created and formed the basis of the synthesis.

#### Narrative synthesis

Due to the anticipated heterogeneity of quantitative data, a narrative synthesis without meta-analysis guidance was employed.^30^

## Results

### Summary of included studies

Twenty-five of the 1649 studies met the inclusion criteria (see Fig. 1). Summary of findings in the included studies are presented in Tables 2 and 3. Eleven studies were cross-sectional,^31–42^ six studies used a mixed-method approach,^43–48^ four studies gathered data from focus groups,^39^^,^^49–51^ three studies were structured interviews^52–54^ and one study was a case-study.^55^ Most of the studies took place in the USA,^31–36^^,^^38–43^^,^^46–53^^,^^55^ except for two studies conducted in the UK,^37^^,^^54^ one in Costa Rica^45^ and one in Canada,^44^ and the majority were completed in the last five years.^31^^,^^32^^,^^36–39^^,^^46–48^^,^^50^^,^^52–54^ Included studies had an average sample size of 425 participants. Quantitative studies examined a larger population on average (*n* = 727), compared to qualitative papers (*n* = 36). Most participants were female (84%), and the average age was 44 years old. Not all studies stated how anthropological data was collected.^53^ Six articles examined parents’ perspectives.^40^^,^^47^^,^^48^^,^^51^^,^^53^^,^^55^ The anthropometric measurements were collected by the experimenter in 71% of studies,^31^^,^^33–35^^,^^38–40^^,^^42^^,^^43^^,^^45–49^^,^^51^ compared to 29% who self-reported.^32^^,^^36^^,^^37^^,^^41^^,^^44^^,^^50^

**Table 2:** Characteristics of studies reporting quantitative data (*n* = 14)

Author and year	Location and economic income level^b^	Study design and number of participants	Participant characteristics^d,e^	Measure of FI	Methods and measures	Key findings				
						Financial cost of food	Geographical access and the food environment	Food practices in the home	Psychological aspects of FI	Driving factors
Ashe *et al.*, 2018	• USA • High	• Cross-sectional • *n* = 4672	• 40+ years • 100% female • Mean BMI not stated • 74% non-Hispanic white; 12% non-Hispanic black; 14% other • Female adults, aged ≥40 year	• US Department of Agriculture household food security scale (18Q). • FS = 0; FI = 1–10 • FI = 21%	Data extracted from National Health and Nutrition Examination Survey 2003–2008 Social Support Index				No statistical difference was observed between FS and FI obese participant’s social support	Availability of social support does not affect obesity levels in the FI population
Boman-Davis *et al.*, 2021	• USA • High	• Cross-sectional • *n* = 1378	• 18+ years • 100% female • Mean BMI not stated • Hispanic 69.9%, White 17.1%, African American 7.8%, American Indian, or Alaska Native 0.3%, Asian 3.2%, Other/two or more races 1.8% • Female Adults with obesity and income which is less than 200% of Federal Poverty Level	• US Department of Agriculture’s Household Food Security Module (6Q) • FS = 0 to 1; FI = 2 to 6 • FI = 49%	Data extracted from California Health Interview Survey: a random digital telephone survey Kessler Psychological Distress (K6) Scale Fruit and vegetables affordability assessed via asking ‘How often are the fresh fruits and vegetables you find in your neighbourhood affordable?...’ Delayed Medical Care (Past Year)	A significant association was found between delaying medical care and women who are obese and FI, compared to those who are FS (*P* = 0.0324) A significant association was found between fruit and vegetables affordability and women who are obese and FI, compared to those who are FS (*P* = 0.0033)			A significant association was found between participants with obesity who were likely to have psychological distress and be FI, compared to those who were FS (aOR 4.63, 95% CI = 2.30–9.32, *P* < 0.0001)	Psychological distress delayed medical care and healthy diet is associated with FI women with obesity
Dressler & Smith, 2015	• USA. • High	• Cross-sectional • *n* = 330	• 18–64 years • 100% female • Mean BMI: 31 kg/m^2^ • African American 40%, American Indian 29%; White 19%; Hispanic 3%, Asian 1%; Other 9% • Adult and qualified for SNAP	• US Department of Agriculture’s Household Food Security Module (6Q) • FS = 0 to 1; FI = 2 to 6 • FI = 63% However, researchers have re-assigned 100% of participants as FI as all are SNAP recipients.	Recruitment took place at various community settings Center for Epidemiological Studies Depression Scale. Nutritional outcomes assessed by food intake recalled over previous 24 hours Emotional eating score			A statistical difference was found between non-depressed and depressed participants in regard to: • Energy (kcal): 1887.7 versus 2162.9 • Fibre (g): 14.9 versus 13.6 • Fe (mg): 13.6 versus 13.7 • Fats, oils and added sugars (servings): 18.9 versus 23.7. (*P* < 0.05)	A statistical difference was found between not-depressed participants (−5.3 ± 6.3) and depressed participants (−1.8 ± 6.5) regarding the Emotional Eating Score, (*P* < 0.05)	
Emery *et al.*, 2015	• USA • High	• Cross-sectional observational • *n* = 100	• 20–78 years • 61% female • Mean BMI: 30 kg/m^2^ • Ethnicity not stated • Community-residing adults	• US Department of Agriculture’s Household Food Security Module (6Q) • FS = 0 to 1; FI = 2 to 6 • FI = % not stated	Recruitment took place via through researchmatch, university-specific research posting website, flyers, and by referral from other participants Home food shelf inventory			A statistical association existed between participants who are obese and FI with fewer sweets (*r* = − 0.28, *P* = 0.047) and less alcohol (*r* = − 0.30, *P* = 0.033) in the home		
Florez *et al.*, 2015	• USA • High	• Cross-sectional • *n* = 639	• 18+ years • 77% female • Mean BMI: 31 kg/m^2^ • Black 91.8%; mixed-black 3.5%; non-black 4.7% • Adults who self-identified as the primary food shopper of the household in ‘food deserts’	• US Department of Agriculture’s Household food security module (18Q) • FS compared to FI was not defined • FI = 40%^a^ However, researchers have re-assigned 100% of participants as FI as all are SNAP recipients	Recruitment occurred by enrolling households, following various community advertisements. Computer-assisted personal interviewing (CAPI) method was used. Patient Health Questionnaire-2 (PHQ-2) (2Q) Two 24-hour dietary recall was used to derive Healthy Eating Index-2005 scores			FI was found to be not associated with dietary quality but was associated with increased BMI (*β* = 1.46, *P* = 0.0239)	A statistical association was found between depressive symptoms and a reduced diet quality (*β* = −1.26, *P* < 0.0001), and increased BMI (*β* = 1.46, *P* = 0.0239)	Decision fatigue regarding food choice, due to reduced income, can result in loss of will power. This is compressed by symptoms of depression, with more starchy/sugary food chosen to try and improve mood, consequently increasing weight gain
Katare *et al.*, 2020	• USA • High	• Cross-sectional • *n* = 1743	• 18+ years • 78% female • Mean BMI: 30 kg/m^2^ • Non-Hispanic White 59%; non-Hispanic black 59%; Hispanic 11%; other 12% • Adults participating in EFNEP or SNAP-Ed programmes and able to speak and understand either English or Spanish	• Researchers have assigned 100% of participants as FI as all are SNAP recipient. • FI = 100%^a^	Recruitment occurred via convenience sampling Effect of Neighbourhood Characteristics on Food Consumption and Health Behaviour survey (adapted)		FI participants who shop at convenience stores most often, were more likely to be obese: aOR: 0.56, 95% CI = 0.30–1.00, *P* < 0.1. There was no statistical association of those who are obese and FI, with living >5 miles away from where the household grocery shopping is conducted; or lack of public transport limiting food store choice	FI participants who are obese are less likely to perceive that there is a large availability of affordable fruit and vegetables, compared to those who are not obese: aOR: 0.79, 95% CI = 0.64–0.98, *P* < 0.05		Perceptions of healthy food might impact obesity levels
Keenan *et al.*, 2021	• UK • High	• Cross-sectional • *n* = 604	• 18–75 years • 90% female • Mean BMI: 29 kg/m^2^ • White 96%, Other 4% • Adults	• US Department of Agriculture Household Food Security (10Q) • FS = 0 to 2; FI = 3 to 10 • FI = 21%	Recruitment took place at food banks, via universities research participation website and Facebook adverts 9 Physical symptoms of stress Yorkshire Health Survey Depression, Anxiety, and Stress Scale (DASS) Allostatic load: elevated blood pressure, blood glucose and/or medication to lower cholesterol Palatable Eating Motives Scale Abbreviated Drinking Motives Questionnaire			There was no indirect relationship between FI and obesity and diet quality (Yorkshire Health Survey)	FI and obesity are indirectly related via increased distress (Physical symptoms and DASS) and eating to cope (Palatable Eating Motives Scale), when controlling for participants income level There was no indirect relationship between FI and obesity and drinking to cope (Drinking motives questionnaire) or Allostatic load	
Marmash *et al.*, 2022	• USA • High	• Cross-sectional • n = 83	• 19+ years • 81% female • Mean BMI Not stated • Hispanic 64%; non-Hispanic white 29%; other 7% • Adults who have attended a Food Bank more than once (100% Food bank users)	• US Department of Agriculture Household Food Security (18Q) • FS = 0 to 2; FI = 3 + • FI = 68% However, researchers have re-assigned 100% of participants as FI as all are SNAP recipients.	Recruitment occurred by convenience sampling. US DGA 2020–2025 recommendations of daily fruit and vegetable consumption			No statistical difference was observed between FI non-obese and Obese individuals (%): • Fruit: 6.1 versus 6.0; • Vegetables: 12.5 versus 10.0; • Fruit and Vegetables: 18.6 versus 16.0		No difference was observed in fruit and vegetable dietary intake of those who are FI and non-obese compared to FI and obese
Papan & Clow, 2015	• Canada • High	• Mixed methods • *n* = 27	• 27–73 years • 100% Female • Mean BMI not stated • Ethnicity not specified • Adults who self-identify as a woman, with a BMI ≥ 25 kg/m^2^ and FI	• Questions were sourced from Canadian Community Health Survey (CCHS) (Cycle 2.2, Nutrition, 2004 and Cycle 3.1, 2005) and First Nations Regional Longitudinal Health Survey (RHS) (Adult Questionnaire, 2008) • FI = 100%	Recruitment took place at various community settings.	Women who are FI and obese,^b^ stated that they often or sometimes could not afford to eat balanced meals.		67% of women who were FI and obese^b^ stated that they had ‘enough, but not always the kinds of food they wanted to eat’, however 30% said they ‘sometimes’ or ‘often’ did not have enough to eat	Women who are FI and obese^b^ agreed that the following statement was often true and sometimes true 85% of the time, ‘Do you or other household members worry that food would run out before you got money to buy more?’	
Price *et al.*, 2019	• USA • High	• Cross-sectional • *n* = 127	• 18+ years • 73% female • Mean BMI: 48 kg/m^2^ • White 67.2%; African American 11.5%; Other Non-white 18.4% • Adults awaiting bariatric surgery	• US Department of Agriculture Household Food Security (10Q) • FS = 0 to 2; FI = 3 to 10 • FI = 31%	Recruited through pre-operative bariatric surgery programme Price et al developed five additional questions to assess FI	52% of those who were FI agreed that ‘When our money runs low, researchers shop at fast-food restaurants to save money’, compared to 13% who are FS 81% of those who were FI agreed that ‘I try to only buy healthy foods, but I end up buying unhealthy foods that are cheaper’, compared to 71% who were FS 48% of those who were FI agreed that ‘In the past 12 months, I found myself purchasing a lot of food at the beginning of the month then running out of food later in the month’, compared to 7% who were FS 45% of those who were FI agreed that ‘I feel that it could be a hardship to purchase needed vitamins, protein shakes or food items recommended by the bariatric surgery programme’, compared to 14% who were FS	42% of those who were FI agreed that ‘I worry about having the time and transportation to buy my groceries’, compared to 4% who were FS			Age was found to be a driver of FI, as those who were younger were more likely to be FI In this specific population, the impact of FI may limit participants recovery from bariatric surgery, due to the financial and geographical access pressures upon them
Richardson *et al.*, 2015	• USA • High	• Cross-sectional • *n* = 101	• 18–44 years • 100% female • Mean BMI: 32 kg/m^2^ ^•^ Non-Hispanic White 33%; Non-Hispanic Black 46%; Hispanic 6% • Adults with a child aged 6 months to 5 years, and a participant of WIC.	• Researchers have assigned 100% of participants as FI as all are WIC recipients • FI = 100%	Recruited eligible participants at WIC clinic visits 14-item Perceived Stress Scale. 18-item Three-Factor Eating Questionnaire assessing eating behaviour Healthy Eating Index-2010, calculated from 24-hour dietary recall.			A statistical difference was found between participants who were severely obese (≥35 kg/m^2^) and diet quality scores, compared to those with a BMI less than 25 kg/m^2^ (*P* = 0.007). However, there was no significance for those participants who were moderately obese (*P* = 0.15)	A significant association existed between severe obesity (*β* = 0.26, *P* = 0.007), emotional eating (*β* = 0.50, *P* < 0.001) and uncontrolled eating behaviours (*β* = 0.38, *P* < 0.001) and perceived stress in low-income women	Factors affecting obesity is different depending on severity of obesity. Richardson *et al*. proposed than lipogenesis caused by higher cortisol levels, may be a potential driver for those who are severely obese and experiencing increased stress
Rogers *et al.*, 2016	• USA. • High	• Cross-sectional • *n* = 116	• 40–70 years • 86% female • FI and Obese: 36 kg/m^2^ • White 50%; African American 48%; mixed 2% • Adult	• One item question (Inglis *et al.*, 2008) ‘Have you ever run out of food in the last 12 months because you could not afford to buy more?’ (1Q) • FS = No; FI = Yes • FI = 19%	Recruitment took place at various community settings. Mental health was assessed via the number of days during the past month when they felt down, stressed, or otherwise impaired by mental health. Food cost assessed via one question adapted from Inglis *et al*. (2008) ‘When you purchase food for you or your family, how important is the following to you? How important is cost?’ Unhealthy food preparation was assessed using 17 items Home environment was assessed by asking about the availability of unhealthy food and drink in the home	A significant association existed between food security status and the importance of food cost *F*(1, 114) = 10.70, *P* = .001, R2 = 0.086		A significant association existed between food security status and unhealthy food preparation, *F*(1, 112) = 3.75, *P* = .055, R2 = 0.032 No significant findings were found for home food environment (*P* > 0.05)	Associations were not significant for mental health, FI and obesity (*P* > 0.05)	
Sharpe *et al.*, 2016	• USA • High	• Cross-sectional • *n* = 202	• 18+ years • 100% female • Mean BMI = 41 kg/m^2^ • African American 87%; White 8%; Mixed 5% • Female adults from low-income neighbourhoods, with BMI > 25 kg/m^2^ and waist circumference ≥ 88 cm	• 12-month Food Security Scale (6Q) • FS = 0–1; FI = 2–6 • FI = 39%	Recruitment occurred by through a community advisory board of women from the relevant neighbourhoods Modified Self-Efficacy Scale Abbreviated Eating Behaviour Patterns Questionnaire Modified Eating Behaviour Patterns Questionnaire Center for Epidemiological Studies Depression Scale (CESD-10) Social Support for Healthy Eating Questionnaire Alternative Healthy Eating Index (AHEI)			Healthy eating behaviour (eating Behaviour Patterns Questionnaire) was statistically significant between those who were FI and obese^c^ (22, SD 6), compared to those who were FS (11, SD 4, *P* = 0.05). AHEI assessed the difference between food quality in FI and FS participants, and found only protein, carbohydrates and lean meat was statistically significantly consumed less (*P* = 0.05, *P* = 0.04, *P* = 0.04, respectively)	FI women who were obese,^c^ had a statistically significant higher depression score (11, SD 6) and emotional eating score (10, SD 3) compared to those who are FS (8, SD 5, *P* = 0.001; 11, SD 4, *P* = 0.02, respectfully) FI women who were obese^c^ had a statistically significant lower self-efficacy for healthy eating score (18, SD 4), compared to those who are FS (19, SD 5, *P* = 0.01) FI women who were obese,^c^ did not differ significantly from FS women, in terms of social support for healthy eating (*P* = 0.1)	The psychological aspects, such as depression, emotional eating and low self-efficacy are the drivers for women who are obese and FI
Taylor *et al.*, 2021	• USA • High	•Mixed methods • *n* = 50	• 18+ years • 50% female • Mean BMI = 31 kg/m^2^ • African American or Black 16%; White 58%; Asian 4%; American Indian or Alaska Native 2%; Other 20% • Parents adults that are FI and speak English, with children aged 2.5 to 10 years that cohabit for ≥4 days a week	• US Department of Agriculture Household Food Security Survey Module (18Q) • FI = affirmative to one of the first four questions • FI = 100%	Recruitment took place at various community settings. Center for Epidemiological Studies Depression Index (CES-D)				No statistically significant associations were found between FI, depression, and BMI based on bivariate Pearson correlations (*P* > 0.05)	

a Studies were re-assigned as FI, as 100% of the population used SNAP.

b Based on the Organisation for Economic Co-operation and Development (OECD) list of high- and low-/middle-income countries.

c Participants were re-classified as obese, as 93% of participants were obese (BMI ≥30 kg/m^2^).

d Participants were re-classified as obese, as 95% of participants were obese (BMI ≥30 kg/m^2^), and they had a mean BMI of 40 kg/m^2^.

e Age range (years); female participants (%); BMI average (kg/m^2^); Ethnicity (%); Key demographics; aOR, adjusted odd’s ratio; BMI, Body Mass Index; CI, confidence interval; DGA, Dietary Guidelines for Americans; EFNEP, Expanded Food and Nutrition Education Program; FI, food insecure; FS, food secure; SD, standard deviation; SNAP, Supplemental Nutrition Assistance Program; SNAP-ed, Supplemental Nutrition Assistance Program-Education; WIC, Special Supplemental Nutrition Program for Women, Infants and Children.

**Table 3:** Characteristics of studies reporting qualitative data (*n* = 13)

Author and year	Location and income level^d^	Study design, methods, and measures	Participant characteristics^e^	Measure of FI	Themes						Driving factors
					Financial cost of food	Geographical access and food environment	Food practices at the home	Experience of food assistance	Psychological aspects and FI	Parent–child relationship	
Cheung *et al.*, 2015, a ^43^	• USA • High	• Mixed methods; • 4 focus groups (3 in English and 1 in Spanish) from random selection of participants who reported FI and had BMI data available	• Age no reported • Gender not reported • Ethnicity reported • 100% FI • BMI not reported	• A screening tool that asked two questions • Participants were considered FI if they answered yes to one question (1) In the past month, was there any day when you or anyone in your family went hungry because you did not have enough money for food? (2) Would you be interested in having someone contact you to talk more about getting food resources for you and your family?	Yes	Yes	Yes	Yes			• Low self-efficacy
Stowers *et al.*, 2020 ^52^	• USA • High	• Semi structured interviews • *n* = 10 • Thematic analysis of individual semi-structured interviews conducted via telephone	• Key stakeholders involved in food banking system in a professional capacity (e.g. food bank board member; food bank executive director; anti-hunger organization leader) • Age not reported • Gender not reported • Ethnicity not reported • BMI not reported	N/A				Yes			• Structural inequality and differential access to social and economic resources
Martinez-Jaikel & Frongillo, 2016 ^45^	• Costa Rica • Low/Middle	• Focus groups and interviews • *n* = 28	• Age range = 30–60 (no mean data) • 100% Female • Ethnicity not stated • 57% Food insecure • BMI not reported	• Costa Rica FI scale	Yes		Yes		Yes	Yes	• Low self-efficacy • Pressures on women
Myers *et al.*, 2020 ^46^	• USA • High	• Mixed methods • *n* = 56 • Thematic analysis of interviews of subsample of participants	• Mean age = 37.8 • 64.7% Female • 64.7% African American • 54% Food insecure • Mean BMI = 35.5 kg/m^2^	• Six-Item Food Security Survey	Yes		Yes	Yes			• Psychological factors
O'Malley *et al.*, 2012 ^55^	• USA • High	• Commentary • *n* = 1 • Commentary using quotes from patient encounter	• Age not given • 100% Female • Ethnicity not stated • Food security not measured • BMI not reported	• Food Security not measured	Yes		Yes	Yes	Yes		• Unemployment
Papan & Clow, 2015^44^	• Canada • High	• Mixed methods • *n* = 27 • Focus groups to identify experiences of FI women with high BMI’s • Feminist, indigenous and participatory methodological frameworks	• Mean age 51.5 • 100% Female • Ethnicity not stated • 100% Food insecure • Mean BMI not given	• FI screening questions • Participants were considered FI if they answered yes to one question (1) In the past 12 months, have you worried that you would not be able to access sufficient, nutritious, and personally acceptable food through normal food channels? (2) In the past 12 months, have you been unable to obtain sufficient, nutritious, and personally acceptable food through normal food channels? • Food security questionnaire with questions from the Canadian Community Health Survey (CCHS) and the First Nations Regional Longitudinal Health Survey (RHS)	Yes	Yes	Yes	Yes	Yes	Yes	• Intergenerational transmission of poverty • Psychological factors • Lack of choice and agency
Taylor *et al.*, 2019 & 2021^47^^,^^48^	• USA • High	• Mixed methods • *n* = 25 • Semi-structured interviews of mothers and fathers	• Mean age = 37.8 • 50% Female • 58% White, non-Hispanic • 100% Food insecure • Mean BMI = 30.75	• USDA Household Food Security Survey Module (HFSSM)	Yes		Yes		Yes	Yes	• Gendered norms and values
Thompson 2018 ^57^	• UK • High	• Ethnographic study with interviews • *n* = 42 • Observations of food banks and semi-structured interviews	• Mean age not given • % Female not given • Ethnicity not given • Food security not measured • Mean BMI not given	• Food Security measure not used • Participants used food banks or worked with food banks in professional capacity	Yes				Yes		• Psychological factors
Wiig & Smith, 2008 ^51^	• USA • High	• Focus groups • *n* = 92 • Focus groups of mothers using food stamps to gain insight into food choices on a low budget	• Mean age = 37 • 100% Female • 51% African American • 87% Food insecure • Mean BMI not given	• Food Security measure not used; use of food stamps was recorded	Yes	Yes	Yes	Yes		Yes	• Food choices on limited budget
Dressler & Smith, 2013,^a^^49^	• USA • High	• Focus groups • *n* = 83 • 16 focus groups of clients of food pantries, neighbourhood centres, soup kitchens and WIC participants	• Ages not given • 100% Female • 58% African American • 100% SNAP recipients • Mean BMI not given	• No of FI • All women discussed experiences of limited access to food	Yes		Yes	Yes	Yes	Yes	• Low self-efficacy • Stress • Emotional eating
Kinsey *et al.*, 2019,^a^^53^	• USA • High	• Interviews • *n* = 18 • In depth individual interviews, three interviews with each participant at the beginning, middle and end of month	• Mean age = 36.5 • 89% Female • 100% African American • 100% SNAP recipients • Mean BMI not given	• Measure not explained • Very low food security was skipping meals (56%)	Yes			Yes			• Chronic illness
Knippen *et al.*, 2020,^b^^50^	• USA • High	• Focus groups • *n* = 55 • Focus groups following a structured guide	• Mean age = 72 • 74.5% Female • 96.4% White • 41.8% Food insecure • Mean BMI = 31 kg/m^2^	• 6 item, FI self-efficacy scale	Yes		Yes	Yes	Yes		• Emotional eating
Martinez-Jaikel & Frongillo, 2016 ^45^	• Costa Rica • Low/Middle	• Focus groups and interviews • *n* = 28	• Age range = 30–60 (no mean data) • 100% Female • Ethnicity not stated • 57% Food insecure • BMI not reported	• Costa Rica FI scale	Yes		Yes		Yes	Yes	• Low self-efficacy • Pressures on women
Myers *et al.*, 2020,^c^^46^	• USA • High	• Mixed methods • *n* = 56 • Thematic analysis of interviews of subsample of participants	• Mean age = 37.8 • 64.7% Female • 64.7% African American • 54% Food insecure • Mean BMI = 35.5 kg/m^2^	• Six-Item Food Security Survey	Yes		Yes	Yes			• Psychological factors
O'Malley *et al.*, 2012 ^55^	• USA • High	• Commentary • *n* = 1 • Commentary using quotes from patient encounter	• Age not given • 100% Female • Ethnicity not stated • Food security not measured • BMI not reported	• Food Security not measured	Yes		Yes	Yes	Yes		• Unemployment
Papan & Clow, 2015, c^44^	• Canada • High	• Mixed methods • *n* = 27 • Focus groups to identify experiences of FI women with high BMI’s • Feminist, indigenous and participatory methodological frameworks	• Mean age 51.5 • 100% Female • Ethnicity not stated • 100% Food insecure • Mean BMI not given	• FI screening questions • Participants were considered FI if they answered yes to one question (1) In the past 12 months, have you worried that you would not be able to access sufficient, nutritious, and personally acceptable food through normal food channels? (2) In the past 12 months, have you been unable to obtain sufficient, nutritious, and personally acceptable food through normal food channels? • Food security questionnaire with questions from the Canadian Community Health Survey (CCHS) and the First Nations Regional Longitudinal Health Survey (RHS)	Yes	Yes	Yes	Yes	Yes	Yes	• Intergenerational transmission of poverty • Psychological factors • Lack of choice and agency
Taylor *et al.*, 2019 & 2021^47^^,^^48^	• USA • High	• Mixed methods • *n* = 25 • Semi-structured interviews of mothers and fathers	• Mean age = 37.8 • 50% Female • 58% White, non-Hispanic • 100% Food insecure • Mean BMI = 30.75	• USDA Household Food Security Survey Module (HFSSM)	Yes		Yes		Yes	Yes	• Gendered norms and values
Thompson 2018 ^57^	• UK • High	• Ethnographic study with interviews • *n* = 42 • Observations of food banks and semi-structured interviews	• Mean age not given • % Female not given • Ethnicity not given • Food security not measured • Mean BMI not given	• Food Security measure not used • Participants used food banks or worked with food banks in professional capacity	Yes				Yes		• Psychological factors
Wiig & Smith, 2008 ^51^	• USA • High	• Focus groups • *n* = 92 • Focus groups of mothers using food stamps to gain insight into food choices on a low budget	• Mean age = 37 • 100% Female • 51% African American • 87% Food insecure • Mean BMI not given	• Food Security measure not used; use of food stamps was recorded	Yes	Yes	Yes	Yes		Yes	• Food choices on limited budget

a Reported with BMI, data extracted only from participants with BMI ≥ 30 (kg/m^2^).

b Data only from quotes explicitly about experience of obesity and FI.

c Cannot separate data from those with overweight and FI versus obesity and FI so data of both included.

d Based on the Organisation for Economic Co-operation and Development (OECD) list of high- and low-/middle-income countries.

e Age range (years); female participants (%); Ethnicity (%); Key demographics; FI (%); BMI average (kg/m^2^); BMI, Body Mass Index; FI, food insecure; SNAP, Nutrition Assistance Program; WIC, Special Supplemental Nutrition Program for Women, Infants and Children.

### Results of quality appraisal

Included studies were appraised according to Greenhalgh^28^ (see Supplementary Tables 2 and 3). Most of the quantitative studies were cross-sectional. Qualitative studies were of good conduct as interviews and focus groups were recorded, transcribed ‘verbatim’ and themes were analysed independently. However, several studies did not specify quality controls, such as continuing the study until content saturation, or calculating Cohen’s Kappa value to investigate inter-rater reliability.^43^^,^^44^^,^^47^^,^^48^^,^^50^^,^^51^ Further, one study was found to be of low rigour.^55^

### Limitations of included studies

Many authors employed oral questionnaires, conducted interviews, or focus groups, which increases the probability conformity bias.^32^^,^^38^^,^^40^^,^^41^^,^^43–45^^,^^47–54^ In addition, a significant limitation was the variability in FI measurements. While 12 studies used a variant of the US Department of Agriculture Household Food Security Scale^56^ employing between 6 and 18 validated questions,^31–35^^,^^37–39^^,^^42^^,^^46–48^^,^^50^ three studies used either one or two of those questions, thus limiting the study generalisability.^41^^,^^43^^,^^44^

Quantitative and qualitative results are summarized in Tables 2 and 3, respectively. The following six themes were identified in the thematic synthesis: (i) financial cost of food; (ii) geographical access and the food environment; (iii) food practices at home; (iv) experience of food assistance; (v) psychological aspects related to FI and (vi) parent–child relationships. These themes have been incorporated into the quantitative results for comparison. No quantitative data was found for ‘experience of food assistance’ or ‘parent–child relationships’.

### Narrative review of quantitative studies

#### Financial cost of food

The cost of food was a common theme in several studies, such that 93% of participants who were obese and FI reported that they ‘often’ or ‘sometimes’ could not afford balanced meals.^44^ Similarly, in people with obesity, a statistically significant difference (*P* = 0.0033) in affordability of fruit and vegetables was identified, whereby fruit and vegetables were less affordable for people with FI compared to those who were food secure.^32^ Price *et al*.^39^ (2019) examined food-secure and FI pre-bariatric surgery participants and identified that 52% of FI used fast-food restaurants to save money, compared to 13% who were food-secure. Finally, there was a significant association between FI people with obesity and delayed medical care, compared to food-secure people with obesity (*P* = 0.0324).^32^

#### Geographical access and the food environment

Two studies examined the geographical access and the food environment. Price *et al.*^39^ found that 42% of individuals who were obese and food-insecure were limited by time and means of transport to purchase food, compared to 4% of those who were obese and food-secure.

Additionally, purchasing food at nearby convenience stores was associated with obesity (adjusted OR 0.56, CI 0.30–1.00; (*P* < 0.1)).^36^ This is despite there being no significant relationship between obesity, FI and the distance from home to grocery stores (adjusted OR 1.18, CI 0.95–1.47).^36^

#### Food practices at home

Food practices at home were thoroughly assessed as a factor influencing participants who are obese and FI. Although dietary intake during FI did not show a clear relationship with obesity (see Table 2), reduced perceived accessibility of affordable fruit and vegetables (adjusted OR 0.79, 95% CI = 0.64–0.98 (*P* < 0.05))^36^ and altered macronutrient intake ^33^^,^^42^ were identified. Specifically, participants who were obese and FI consumed less protein and increased carbohydrate levels, compared to food-secure participants (*P* = 0.05 and *P* = 0.04, respectively).^42^ Rogers *et al.* also identified that FI was significantly associated with unhealthy food preparation (*F*(1, 112) = 3.75, *P* = 0.055, R2 = 0.032), but not the home food environment (*P* > 0.05).^41^

However, in one study, women who were severely obese were more likely to have a greater diet quality score than those that were a normal weight (*β* = 0.25, *P* = 0.007), as defined by the Healthy Eating Index 2010.^40^ Although the authors hypothesize that eating above recommended caloric intake could result in a higher diet quality score, further studies found no relationship between food practices, FI and obesity.^37^^,^^38^

#### Psychological aspects related to FI

Seven authors found statistically significant relationships between FI, obesity and mental health,^32^^,^^33^^,^^35^^,^^37^^,^^40^^,^^42^^,^^44^ with few exceptions.^31^^,^^41^^,^^47^ Psychological stress was significantly more common among people with obesity and FI compared to those that were obese and food-secure with an adjusted odds ratio of 4.63 (95% CI = 2.30–9.32).^32^ Richardson *et al*.^40^ found that severe obesity (*β* = 0.26, *P* = 0.007), emotional eating (*β* = 0.50, *P* < 0.001) and uncontrolled eating behaviours (*β* = 0.38, *P* < 0.001) were all significantly associated with perceived stress in low-income women.

Depression was found to be more significant in FI women with obesity compared to food-secure women with obesity (*P* = 0.001).^42^ Similarly, 85% of participants marked ‘true’ or ‘sometimes true’ to the statement ‘Do you or other household members worry that food would run out before you got money to buy more’.^44^ Furthermore, eating to cope was indirectly linked to FI and obesity, however, drinking to cope was not.^37^

### Thematic synthesis

#### Financial cost of food

The cost of food was the most common theme identified. The high cost of healthy foods and low-cost of unhealthy, processed foods were frequently cited as reasons for excess weight and unhealthy eating patterns.^43^^,^^45^^,^^46^^,^^49^^,^^51^^,^^53^ Comments such as ‘We don’t have enough money to buy healthy food’^44^ featured heavily. Healthy food such as fruit and vegetables were considered ‘a luxury that, that’s for someone else’*.*^44^ The cost of food was considered the most important factor when making food purchasing decisions: ‘“First and foremost for me, well if I’m shopping for food, it’s costs, and then taste. What can I make that’s cheap and tastes good” (BMI 40.4)’.^49^

Paying for household bills and medicines left little money for food.^44^^,^^54^ Strategic choices were made when shopping to purchase foods that would last, lead to satiety, and could be used for multiple meals.^44^^,^^45^^,^^51^ Medically appropriate diets were considered unaffordable. ‘I just can’t go out and afford to buy all the proper foods that we’re supposed to eat’^44^, which led to ‘diet trade off’s and skipping meals’.^53^

Notions that incomes and government assistance programmes are not enough to meet the cost of living or a healthy diet, were common.^43^ ‘I mean, they tell you to have these fruit and vegetables, but I can’t afford them!’.^44^ Addressing the cost of food was considered the ‘number one factor’^44^ that would enable a healthier diet.

#### Geographical access and the food environment

The cost of travel to shops was a barrier to healthy eating for participants who lived further away from supermarkets, while paying for transport reduced people’s food budget even further.^43^^,^^44^ Challenges related to mobility issues due to chronic illness or obesity were also raised. As one person explains, ‘that’s twenty bucks that I can’t spend on groceries because I’ve got to spend it on a cab because I can’t walk that distance. Because of my weight, because of my mobility issues, I just can’t do it’.^44^

While many participants reported going to multiple shops to use their food stamps, choosing where they considered cheapest or where they could buy in bulk, a lack of transport options meant others had to shop wherever was closest.^51^

#### Food practices at home

Some participants reported cooking frequently, ‘“I cook every day, trust me” (BMI, 33.4)’*.*^49^ Other participants had little interest in cooking healthy food, because they felt it was more time consuming, difficult, expensive and not as palatable.^45^^,^^55^ Reasons for eating out or eating convenience meals included having ‘“no time for cooking” (BMI > 30 kg/m^2^)’^43^ or a dislike of cooking.^43^^,^^55^ Practices to avoid food waste and make food last longer were also mentioned, such as diluting milk with water.^43^^,^^51^ Some quotes highlighted the stresses faced by people which meant they were ‘thinking about how to get by day to day… [and not about] planning meals…’*.*^46^ One paper discussed binge eating when food was available due to not knowing when the next meal may be.^48^

#### Experience of food assistance

Participants across studies expressed it was difficult to find culturally or nutritionally appropriate food in food banks and considered it unhealthy.^43^^,^^44^^,^^50^^,^^52^^,^^53^^,^^57^ The quality of the food at food banks was also questioned by some recipients.^44^

#### Psychological aspects related to FI

The stresses of living with FI were evident.^45^^,^^47^^,^^49^^,^^54^ Participants were ‘“always worried about food” (BMI, 49.6)’*.*^49^ References to emotional eating or a loss of control when eating due to low mood and anxiety were common.^44^^,^^45^^,^^47^^,^^50^ ‘When I stress a lot, I, I feel like I tend to eat more… to kind of like, comfort myself’. Another participant described panic attacks due to the stress of FI.^47^

Stigma or embarrassment about going to food banks was discussed in two studies.^44^^,^^55^ ‘And the stigma – going to the food bank is an awful stigma in itself. Standing out there in a line and having somebody determine when you’re poor’.^44^ Low self-esteem and social isolation among participants was also identified, as were concurrent mental health issues.^44^^,^^45^^,^^54^

#### Parent–child relationships

Parents sacrificing their food so children could eat was a common theme.^44^^,^^47–49^ ‘I feed them and then I eat… I mean, they’re more important than I am’.^44^ The majority of such responses came from mothers.^44^^,^^47^ However, some fathers also considered it part of their role as ‘parent and provider’.^47^ Some parent’s food purchasing decisions were influenced by children’s preferences, to prevent wasting money, while others described their children as not being fussy, ‘“you hungry, you got to eat” (BMI 49.6)’.^49^

## Discussion

This review highlights the challenges faced by people living with obesity and FI while providing insight into potential driving factors of this relationship. Several major themes emerged, including the financial cost of healthy food, compared to cheaper, convenient, processed foods and psychological factors, such as depression, low self-efficacy and stress. Florez *et al.*^35^ suggested that these themes interact due to increased decision fatigue from constantly adapting diet to fit with food assistance programmes, food availability and quality, leading to depressive symptoms and selection of starchy/sugary foods to combat such emotions. In parallel, Richardson *et al.*^40^ suggested that high cortisol levels from associated FI stress may cause lipogenesis, perpetuating weight gain.

This is consistent with findings from a recent systematic review and meta-ethnography of the experiences of living with obesity, which found that perceptions of stigma were prevalent, while low self-esteem and emotional distress were considered as a cause and effect of living with obesity.^58^ It may be therefore that mental wellbeing is not only negatively affected by the circumstances faced by individuals with obesity who are FI but, may also play a role in the development of both states.

### Implications for practice and policy

The link between FI and obesity is well established.^10^ This review has identified that a lack of access to healthy foods and unhealthy eating behaviours due to FI may contribute to poor physical and mental health among people with obesity. While explanations for obesity are often reduced to behaviour at the individual level, it is essential to recognize the role of the food environment as the social determinant of health, providing the context in which food decisions are made.

Knight and Fritz^59^ argue it is an ethical obligation to ask patients if they are food insecure. There is currently no national measure of FI in the UK, thus a nationally accepted and validated food security measure, such as in USA, would be valuable.^57^ Without identifying and acknowledging the difficulties people with obesity may be experiencing regarding food access, it may not be possible to support them to improve their health in other areas.

In terms of wider implications for society, this review has provided further evidence that a significant underlying contributing factor to people’s food behaviours is the low cost of unhealthy processed foods, compared to more expensive healthier foods. For the poorest 10% of households, 75% of disposable household income would need to be spent on food purchases to meet national dietary guidelines.^60^ Taking the lowest 50% of income deciles together, 30% of disposable income would need to be spent on food, this compares to 12% for the highest 50% of deciles.^60^

Food banks and other assistance programmes provide for people in crisis, but the food provided is not necessarily supportive of a healthy diet.^9^^,^^10^ While these services are a lifeline for many, the issue of FI is often chronic and cannot be resolved with emergency food parcels.^10^ From a social determinants’ perspective, systems change is needed to address the cost of food to ensure that families can afford the food they need to support health, as summarized by Loopstra.^61^

### Strengths and limitations

To our knowledge, this is the first systematic review analysing the life experience of people who are obese and food insecure. However, it is not without its limitations. Researchers assumed that food assistance recipients (food banks, Supplemental Nutrition Assistance Program or Special Supplemental Nutrition Program for Women, Infants and Children) were automatically food insecure, due to lack of measures. Yet, Florez *et al.*^35^ identified that users of food assistance programmes are not always food insecure, nevertheless, it was deemed as an acceptable proxy for the target population.

Furthermore, the review is limited due to the use of only English language and retrieval of papers mostly from the USA (*n* = 22). Only one paper was retrieved from a low-middle income country.^45^ The dominance of North American research in this field may in part be due to frequent monitoring with a standardized measure of FI,^61^ unlike the UK, limiting its generalisability.

## Conclusion

This review aimed to identify the experiences of people who are obese and food insecure. The results of the thematic synthesis provided insight into the hardships faced at the intersection of FI and obesity. These results were echoed by the findings of quantitative studies that highlighted the cost of healthy food as a barrier and the relationship between FI, obesity and adverse mental health outcomes. Together the results, grouped by third-order constructs, provide a wealth of insight into home practices, mental wellbeing and drivers of FI and obesity. In the current context of the rising cost of living, these findings have important implications for society. Food security is a social determinant of health, a systems approach is necessary to elicit policy change that will stem the tide of FI and obesity.

## Supplementary Material

2023_08_14_appendix1searchstrategyforeachdatabase_fdae016

2023_08_14_appendix2quantitativequalityappraisal_fdae016

appendix3qualitativequalityappraisal_fdae016

## Notes

**Rebecca Briggs,** Medical Student

**Hope Rowden,** Medical Student

**Lukasz Lagojda,** CEBIS Specialist

**Timothy Robbins,** Consultant Endocrinologist

**Harpal S. Randeva,** Consultant Endocrinologist

## References

[1] Purdam K , Garratt EA, Esmail A. Hungry? Food insecurity, social stigma and embarrassment in the UK. Sociology 2016;50:1072–88.

[2] FAO, IFAD, UNICEF et al. The State of Food Security and Nutrition in the World. Transforming food systems for food security, improved nutrition and affordable healthy diets for all. FAO: Rome. (2022). https://www.fao.org/3/cc0639en/online/cc0639en.html 10.4060/cc0639en.

[3] FAO, IFAD, UNICEF et al. In: 10.4060/cc0639en (ed). The State of Food Security and Nutrition in the World 2022. Repurposing Food and Agricultural Policies to Make Healthy Diets More Affordable. FAO: Rome, 2022. https://www.fao.org/3/cc0639en/online/cc0639en.html.

[4] Arenas DJ , Thomas A, Wang J. et al. A systematic review and meta-analysis of depression, anxiety, and sleep disorders in US adults with food insecurity. J Gen Intern Med 2019;34:2874–82. 31385212 10.1007/s11606-019-05202-4 PMC6854208

[5] te Vazquez J, Feng SN, Orr CJ. et al. Food insecurity and cardiometabolic conditions: a review of recent research. Curr Nutr Rep 2021;10:243–54. 34152581 10.1007/s13668-021-00364-2 PMC8216092

[6] Thomas MK , Lammert LJ, Beverly EA. Food insecurity and its impact on body weight, type 2 diabetes, cardiovascular disease, and mental health. Curr Cardiovasc Risk Rep 2021;15:15. 34249217 10.1007/s12170-021-00679-3 PMC8255162

[7] Robinson E . Obesity and the cost of living crisis. Int J Obes (Lond) 2022;47:93–4. 36456646 10.1038/s41366-022-01242-9 PMC9715406

[8] Trussel Trust. Emergency Food Parcel Distribution in the United Kingdom: April – September 2022. Trussel Trust: London, (2022). https://www.trusselltrust.org/wp-content/uploads/sites/2/2022/11/MYS-UK-Factsheet-2022.pdf.

[9] Bazerghi C , McKay FH, Dunn M. The role of food banks in addressing food insecurity: a systematic review. J Community Health 2016;41:732–40. 26728281 10.1007/s10900-015-0147-5

[10] Oldroyd L , Eskandari F, Pratt C. et al. The nutritional quality of food parcels provided by food banks and the effectiveness of food banks at reducing food insecurity in developed countries: a mixed-method systematic review. J Hum Nutr Diet 2022;35:1202–29. 35112742 10.1111/jhn.12994 PMC9790279

[11] Kaiser ML , Cafer A. Understanding high incidence of severe obesity and very low food security in food pantry clients: implications for social work. Soc Work Public Health 2018;33:125–39. 29297775 10.1080/19371918.2017.1415181

[12] Dietz WH . Does hunger cause obesity? Pediatrics 1995;95:766–7. 7724321

[13] Frongillo EA , Bernal J. Understanding the coexistence of food insecurity and obesity. Curr Pediatr Rep 2014;2:284–90.

[14] Dinour LM , Bergen D, Yeh M-C. The food insecurity-obesity paradox: a review of the literature and the role food stamps may play. J Am Diet Assoc 2007;107:1952–61. 17964316 10.1016/j.jada.2007.08.006

[15] Farrell P , Thow AM, Abimbola S. et al. How food insecurity could lead to obesity in LMICs: when not enough is too much: a realist review of how food insecurity could lead to obesity in low- and middle-income countries. Health Promot Int 2018;33:812–26. 28541498 10.1093/heapro/dax026

[16] Moradi S , Mirzababaei A, Dadfarma A. et al. Food insecurity and adult weight abnormality risk: a systematic review and meta-analysis. Eur J Nutr 2019;58:45–61. 30219965 10.1007/s00394-018-1819-6

[17] Nettle D , Andrews C, Bateson M. Food insecurity as a driver of obesity in humans: the insurance hypothesis. Behavior Brain Sci 2017;40:e105. 10.1017/S0140525X16000947 PMC5266557 27464638

[18] Brown AGM , Esposito LE, Fisher RA. et al. Food insecurity and obesity: research gaps, opportunities, and challenges. Transl Behav Med 2019;9:980–7. 31570918 10.1093/tbm/ibz117 PMC6937550

[19] Dolezal L , Spratt T. Fat shaming under neoliberalism and COVID-19: examining the UK’s tackling obesity campaign. Sociol Health Illn 2022;45:3–18. 36178389 10.1111/1467-9566.13555 PMC7614026

[20] Pirie I . Disordered eating and the contradictions of neoliberal governance. Sociol Health Illn 2016;38:839–53. 26896419 10.1111/1467-9566.12408

[21] Farrell AE . Fat Shame: Stigma and the fat body in American culture. New York: New York University Press, 2011.

[22] Meulman MA . Sizeism in therapy: fat shaming in supervision. Women Ther 2019;42:156–63.

[23] Page MJ , McKenzie JE, Bossuyt PM. et al. The PRISMA 2020 statement: an updated guideline for reporting systematic reviews. BMJ 2021;372:1–9. 10.1136/bmj.n71. PMC8005924 33782057

[24] Stern C , Jordan Z, McArthur A. Developing the review question and inclusion criteria. Am J Nurs 2014;114:53–6. 10.1097/01.NAJ.0000445689.67800.86 24681476

[25] Greenbaum, S. & T. Contemporary Financial Intermediation. in (eds. Greenbaum, S. I., Thakor, A. V & Boot, A. W. A.) 331–352 ( Academic Press, 2016). 10.1016/B978-0-12-405196-6.00014-8.

[26] Ouzzani M , Hammady H, Fedorowicz Z. et al. Rayyan—a web and mobile app for systematic reviews. Syst Rev 2016;5:210. 27919275 10.1186/s13643-016-0384-4 PMC5139140

[27] Thomas J , Brunton J, Graziosi S. EPPI-Reviewer 4: Software for Research Synthesis. London: Social Science Research Unit, UCL Institute for Education, 2010.

[28] Greenhalgh T . How to Read a Paper: The Basics of Evidence-Based Medicine and Healthcare. Chichester: John Wiley & Sons Ltd, 2019.

[29] Thomas J , Harden A. Methods for the thematic synthesis of qualitative research in systematic reviews. BMC Med Res Methodol 2008;8:45. 18616818 10.1186/1471-2288-8-45 PMC2478656

[30] Campbell M , McKenzie JE, Sowden A. et al. Synthesis without meta-analysis (SWiM) in systematic reviews: reporting guideline. BMJ 2020;368:1–6. 10.1136/bmj.l6890. PMC7190266 31948937

[31] Ashe KM , Lapane KL. Food insecurity and obesity: exploring the role of social support. J Womens Health 2018;27:651–8. 10.1089/jwh.2017.6454 29182494

[32] Boman-Davis M , Jimenez JA, Yokum S. Food insecurity and likely psychological distress: isolation of BMI and income among women in California. J Hunger Environ Nutr 2021;16:95–108.

[33] Dressler H , Smith C. Depression affects emotional eating and dietary intake and is related to food insecurity in a Group of Multiethnic, low-income women. J Hunger Environ Nutr 2015;10:496–510.

[34] Emery CF , Olson KL, Lee VS. et al. Home environment and psychosocial predictors of obesity status among community-residing men and women. Int J Obes (Lond) 2015;39:1401–7. 25916909 10.1038/ijo.2015.70 PMC4632497

[35] Florez KR , Dubowitz T, Ghosh-Dastidar M. et al. Associations between depressive symptomatology, diet, and body mass index among participants in the supplemental nutrition assistance program. J Acad Nutr Diet 2015;115:1102–8. 25769748 10.1016/j.jand.2015.01.001 PMC4484316

[36] Katare B , Lynch K, Savaiano D. Perceived neighbourhood food environment and overweight and obesity among supplemental nutrition assistance program-education (SNAP-Ed) participants in the Midwest US. Public Health Nutr 2021;24:729–37. 10.1017/S136898002000155X PMC11574820 32744206

[37] Keenan GS , Christiansen P, Hardman CA. Household food insecurity, diet quality, and obesity: an explanatory model. Obesity (Silver Spring) 2021;29:143–9. 33135388 10.1002/oby.23033

[38] Marmash D , Ha K, Sakaki JR. et al. The association between diet quality and health status in mobile food pantry users in Northeastern Connecticut. Nutrients 2022;14:1302. 35334959 10.3390/nu14061302 PMC8955894

[39] Price JA , Zickgraf HF, Rigby A. Food insecurity in a pre-bariatric surgery sample: prevalence, demographics and food shopping behaviour. Public Health Nutr 2019;22:2756–65. 31213214 10.1017/S1368980019001320 PMC7057552

[40] Richardson AS , Arsenault JE, Cates SC. et al. Perceived stress, unhealthy eating behaviors, and severe obesity in low-income women. Nutr J 2015;14:122. 26630944 10.1186/s12937-015-0110-4 PMC4668704

[41] Rogers BG , Kegler MC, Berg CJ. et al. Understanding the food insecurity and obesity relationship by examining potential mediators: an exploratory analysis. J Hunger Environ Nutr 2016;11:195–209.

[42] Sharpe PA , Whitaker K, Alia KA. et al. Dietary intake, behaviors and psychosocial factors among women from food-secure and food-insecure households in the United States. Ethn Dis 2016;26:139–46. 27103763 10.18865/ed.26.2.139 PMC4836893

[43] Cheung HC . et al. Food insecurity and body mass index: a longitudinal mixed methods study, Chelsea, Massachusetts, 2009-2013. Prev Chronic Dis 2015;12:E125. 26247425 10.5888/pcd12.150001 PMC4565511

[44] Papan AS , Clow B. The food insecurity—obesity paradox as a vicious cycle for women: inequalities and health. Gend Dev 2015;23:299–317.

[45] Martinez-Jaikel T , Frongillo EA. Primary role of discouragement in coexistence of food insecurity and excess weight in costa Rican women. J Hunger Environ Nutr 2016;11:210–26.

[46] Myers CA , Beyl RA, Martin CK. et al. Psychological mechanisms associated with food security status and BMI in adults: a mixed methods study. Public Health Nutr 2020;23:2501–11. 32597739 10.1017/S1368980020000889 PMC7483214

[47] Taylor EA , Foster JS, Mobley AR. Examining factors related to the food insecurity-obesity paradox in low-income mothers and fathers. Food Nutr Bull 2021;42:309–16. 34002624 10.1177/03795721211011133

[48] Taylor EA , Foster JS, Mobley AR. A qualitative investigation of body weight and weight loss-related attitudes of mothers and fathers in the context of food insecurity. Eat Weight Disord 2019;25:1663–9. 31691198 10.1007/s40519-019-00804-7

[49] Dressler H , Smith C. Health and eating behavior differs between lean/normal and overweight/obese low-income women living in food-insecure environments. Am J Health Promot 2013;27:358–65. 23398131 10.4278/ajhp.120119-QUAL-55

[50] Knippen KL , Lee H, Ford T. et al. “Bad enough to cook for two, worse for one” – mixed method evaluation of eating behavior among community dwelling older adults. J Nutr Gerontol Geriatr 2020;39:214–35. 32352345 10.1080/21551197.2020.1759478

[51] Wiig K , Smith C. The art of grocery shopping on a food stamp budget: factors influencing the food choices of low-income women as they try to make ends meet. Public Health Nutr 2009;12:1726–34. 19068150 10.1017/S1368980008004102

[52] Stowers KC . et al. The hunger-obesity paradox: exploring food banking system characteristics and obesity inequities among food-insecure pantry clients. PloS One 2020;15:e0239778. 33085685 10.1371/journal.pone.0239778 PMC7577435

[53] Kinsey EW , Dupuis R, Oberle M. et al. Chronic disease self-management within the monthly benefit cycle of the supplemental nutrition assistance program. Public Health Nutr 2019;22:2248–59. 31104648 10.1017/S1368980019001071 PMC6641996

[54] Thompson C , Smith D, Cummins S. Understanding the health and wellbeing challenges of the food banking system: a qualitative study of food bank users, providers and referrers in London. Soc Sci Med 2018;211:95–101. 29933211 10.1016/j.socscimed.2018.05.030 PMC6079189

[55] O’Malley JA , Peltier CB, Klein MD. Obese and hungry in the suburbs: the hidden faces of food insecurity. Acad Pediatr 2012;12:163–5. 22583630 10.1016/j.acap.2012.03.042

[56] USDA . Survey Tools, Vol. 2022 USDA: Alexandria. 2022. Preprint at https://www.ers.usda.gov/topics/food-nutrition-assistance/food-security-in-the-u-s/survey-tools/.

[57] Smith D , Thompson C, Harland K. et al. Identifying populations and areas at greatest risk of household food insecurity in England. Applied Geography 2018;91:21–31. 29915447 10.1016/j.apgeog.2017.12.022 PMC6003598

[58] Farrell E , Hollmann E, le Roux CW. et al. The lived experience of patients with obesity: a systematic review and qualitative synthesis. Obes Rev 2021;22:e13334. 34402150 10.1111/obr.13334

[59] Knight JK , Fritz Z. Doctors have an ethical obligation to ask patients about food insecurity: what is stopping us? J Med Ethics 2022;48:707–11. 10.1136/medethics-2021-107409 PMC9554025 34261802

[60] Scott, C., Sunderland, J. & Tylor, A. Affordability of the UK’s Eatwell Guide. The Food Foundation: London, (2018). https://foodfoundation.org.uk/sites/default/files/2021-10/Affordability-of-the-Eatwell-Guide_Final_Web-Version.pdf.

[61] Loopstra R . Interventions to address household food insecurity in high-income countries. Proceedings of the Nutrition Society 2018;77:270–81. 29580316 10.1017/S002966511800006X

