# Supplementary material for: The lived experience of food insecurity among adults with obesity: a quantitative and qualitative systematic review
Source: J Public Health (Oxf). 2024 Feb 26;46(2):230–49. doi: 10.1093/pubmed/fdae016 (PMC11141780; doi:10.1093/pubmed/fdae016)
Supplement: 2023_08_14_appendix1searchstrategyforeachdatabase_fdae016 [file 2023_08_14_appendix1searchstrategyforeachdatabase_fdae016.docx]

**Supplementary Table 1.** Search strategy for MEDLINE, EMBASE, AMED and APA PsychInfo *via* Ovid.

1 obes*.mp. or Obesity/

2 food poverty.mp.

3 food crisis.mp.

4 cost of food.mp.

5 Food Insecurity/ or food insecure.mp.

6 food pantry.mp.

7 food bank.mp.

8 food cost.mp.

9 food insecurity.mp. or Food Insecurity/

10 food poverty.mp.

11 low food security.mp.

12 2 or 3 or 4 or 5 or 6 or 7 or 8 or 9 or 10 or 11

13 1 and 12

14 limit 13 to (yr="2007 -Current" and english)

15 obes*.mp. or Obesity/

16 Poverty/ or poverty.mp.

17 Food/ or food.mp.

18 15 and 16 and 17

19 low socioeconomic status.mp.

20 Low SES.mp.

21 19 or 20

22 15 and 17 and 21

23 hunger obesity paradox.mp.

24 15 and 23

25 cost of living.mp.

26 15 and 25

27 14 or 18 or 22 or 24 or 26

28 limit 27 to (yr="2007 -Current" and english)

Search strategy adapted for ASSIA:

| 1 Obes* AND (food poverty OR food crisis OR cost of food OR food bank OR food insecure OR food pantry OR food cost OR food insecurity OR low food security) |
| --- |
| OR |
| 2 Obes* AND (poverty AND food) |
| OR |
| 3 Obes* AND (low socioeconomic status OR low SES) AND food |
| OR |
| 4 Obes* AND hunger-obesity paradox |
| OR |
| 5 Obes* AND cost of living |

Search strategy adapted Web of Science:

| (‘obes*’) AND ((‘Food insecurity’ OR ‘food security’ OR ‘food bank’ OR ‘food deserts’) AND (‘poverty’ OR ‘low socioeconomic status’ OR ‘cost of living’)) OR (‘hunger obesity paradox’) |
| --- |
