# Supplementary material for: The lived experience of food insecurity among adults with obesity: a quantitative and qualitative systematic review
Source: J Public Health (Oxf). 2024 Feb 26;46(2):230–49. doi: 10.1093/pubmed/fdae016 (PMC11141780; doi:10.1093/pubmed/fdae016)
Supplement: 2023_08_14_appendix2quantitativequalityappraisal_fdae016 [file 2023_08_14_appendix2quantitativequalityappraisal_fdae016.docx]

**Supplementary Table 2:** Quality appraisal of quantitative studies.

| Author, Date | 1)  Aim and appropriateness of the Method? | 2)  Was a previously published and validated questionnaire used? | 3i)  Justified claims about validity | 3ii)  Justified claims about reliability | 4) Presented well, aspects considered | 5) Instructions and explanations | 6)  Piloted? | 7) Sample selection and representative? | 8)  How was survey distribute? | 9)  Were needs of subgroups considered? | 10) Response rate? | 11)  What analysis was carried out and was it appropriate | 12) Were non-significant results reported? | 13)  Use of qualitative data and appropriate analysis? | 14) Appropriate link between data and conclusions? |
| --- | --- | --- | --- | --- | --- | --- | --- | --- | --- | --- | --- | --- | --- | --- | --- |
| Ashe *et al*., 2018 | Understanding the role of social support with those who are obese and FI | Yes, all data is from previously published data | Validity was mentioned, but not explicitly for Social Support questions. However, all data is from previously published survey. | Reliability was not mentioned for the survey; however, data was from previously published data. | N/A | N/A | N/A | Yes | N/A | N/A | N/A | Yes  Adjusted odds ratios and stratified logistic regression was reported. | Yes | N/A | Yes |
| Boman-Davis *et al*., 2021 | Understanding the role of psychological distress in those who are obese and FI | Yes, all data is from previously published data | Validity was not mentioned, but all data is from a previously published survey. | Reliability was not mentioned, but all data is from a previously published survey. | N/A | N/A | N/A | Yes, but just from one American state. | Yes Random digit-dial telephone survey | Yes Survey conducted in six languages | Not stated | Yes  Bivariate analysis and multiple logistic regression were recorded. Utilised SAS Studio V3.7 | Yes | N/A | Yes |
| Dressler & Smith, 2015 | Understanding how emotional eating and dietary intake is related to FI | Yes | Yes | Yes | Yes | Not stated | Not stated | Yes, but just from one American state. | In-person, at various community settings | Not stated | Not stated | Statistical Package for the Social Sciences V17.0.  *t*-tests, analyses of variance, chi-square and Mann-Whitney tests, Multiple linear regressions and Sobel tests were recorded. | Yes | N/A | Yes |
| Emery *et al.,* 2015 | Yes. Understanding how community-residing obese and non-obese participants differ in their home environment and psychological aspects, as predictors of obesity. | Yes | Yes, except for Food insecurity; HADS; ESE; Medical Outcomes. | Yes | Yes | Not stated | N/A | Yes | Did not state how survey was distributed. | Not stated | Yes  100% | Yes  Data were analysed with *t*-tests, chi-square tests and analysis of variance comparing BMI groups (obese vs nonobese) across all dimensions and presented in paper. | Yes | N/A | Yes |
| Florez *et al*., 2015 | Yes. Researchers wanted to understand how diet, BMI and depression are interlinked | Yes | Validity was not mentioned for any surveys, but all surveys are previously published. | Reliability was assessed for PHQ-2 via Cronbach's alpha (0.54), but this is lower than normally accepted values (0.5-0.6 suggest 'poor' reliability). Cronbach's alpha was not mentioned for 18-item USDA Household Food Security Survey, or HEI-2005. | Yes | Yes Computer-assisted personal interviewing method (CAPI) | N/A | Yes | Yes, Survey used CAPI, thus was researcher led | Yes  Recruitment took place by people who lived in the same area | Yes  87% | Yes  Two separate multivariate OLS regressions were modelled for depressive symptomatology for each of the weight-related outcomes, while controlling for sociodemographic factors. | Yes | N/A | Yes |
| Katare *et al.,* 2021 | Yes. Understanding the perceptions of SNAP-ed and EFNEP users, who are overweight or obese. | Yes, except Physical symptoms of stress and Allostatic load | Yes | No, reliability was not mentioned. | Yes | Not stated | Yes  The survey was piloted through the Nutritional Education Program (n = 66). | Yes | Not stated | Yes  The survey was written in English and Spanish | Not stated | Yes  Logistic regression. Analysis was carried out on STATA V15 | Yes | N/A | Yes |
| Keenan *et al.,* 2021 | Yes. Understanding the impact of food insecurity on diet quality, obesity and physical and psychological stress | Yes, except Physical symptoms of stress and Allostatic load | Yes, except it was not mentioned for USDA FI, DASS, Physical Symptoms of stress or Allostatic Load. | Yes, except it was not mentioned for USDA FI or Allostatic Load. | Yes | Yes, Questionnaire was hosted online via Qualtrics | NA | Yes | Yes, Survey was distributed online via Qualtrics. | Not stated | Yes  80% | Yes  Analysis was performed on Mplus. Secondary analysis to control for income. | Yes | N/A | Yes |
| Marmash *et al.,* 2022 | Yes. Understanding how those who use mobile food banks affect diet and health status | Yes | Validity was not mentioned for any surveys, but all surveys were previously published. | Reliability was not mentioned for any surveys, but all surveys are previously published. | Yes | Yes, Questionnaires were administered orally by research staff | NA | Yes | Yes, distributed from researchers orally. | Not stated | Yes  100% | Yes  Wilcoxon rank-sum test and Kruskal–Wallis test was used due to the small sample size.  Data analysis was conducted using SAS software | Yes | N/A | Yes |
| Price *et al.,* 2019 | Yes.  FI and bariatric patients | Yes | Yes | Yes | Yes | Yes  Bariatric psychologist available to support | Not stated | Yes | Yes, at medical centre | Yes Psychologist to support literacy | Yes (>=90%) | Yes  IBM SPSS Statistics V25.0 was utilised. They performed χ^2^ analyses for eight discrete binary variables. Cramer’s V effect sizes were produced for analyses. Used one-way ANOVA, non- parametric Spearman’s ρ. | Yes | N/A | Yes |
| Richardson *et al*., 2015 | Yes. Understanding the role of stress in obesity and FI | Yes | Yes | Yes | Yes | Yes Guided completion via interviewer | Not stated | Yes | Yes | Not stated | Not stated | Yes  Stata 13.0 and Mplus V7.11  was utilised.  Structural equation modelling was used for analysis. | Yes | N/A | Yes |
| Rogers  *et al.,* 2016 | Yes. Understanding potential mediators between FI and obesity. | No, except Unhealthy food preparation was assessed internally via Cronbach alpha. FI, Food Cost and Mental Health were measured via one question. FI and Food Cost used a question which was previously used in another publication. FI could have been assessed by the more frequently used USDA Food Security Survey.   Home food environment and Eating Out 'where asked questions', but no greater detail was given. | No. Validity was not mentioned for any surveys. | Yes, except FI, Food Cost, Mental Health, Home Food Environment and Eating Out did not mention reliability. | Yes | Yes Survey was completed via phone interview. | Not stated | Yes | Yes, survey was completed through phone interview with participants. | Not stated | Yes  95% | Yes  Bivariate analysis, Multiple linear regression and Sobel's tests. | Yes | N/A | Yes |
| Sharpe *et a*l., 2016 | Yes, dietary intake differences but 24h dietary recall can be limited | Yes | Yes | Yes | Yes | Yes Survey was completed via phone interview | Not stated | Yes | Yes | Not stated | Yes  99% | Yes  Statistical Analysis System 9.2 computed means, standard deviations, frequencies, and percentages. T-tests and chi-squared tests (or Fisher’s exact test for cell sizes <5) were utilised. | Yes | N/A | Yes, however more could have been explored relating to psychology and food behaviour outcomes |

Quantitative Analysis Appraisal. Based on Greenhalgh (2019) principals for quality appraisal. DASS = Depression Anxiety Stress Scales; EFNEP = Expanded Food and Nutrition Education Program; ESE = Eating Self-Efficacy Scale; FI = Food insecurity; HADS = Hospital Anxiety and Depression Scale; HEI-2005 = Healthy Eating Index-2005; PHQ-2 = Patient Health Questionnaire–2; SNAP = Supplemental Nutrition Assistance Program; USDA = United Stated Department of Agriculture.
