# Supplementary material for: The lived experience of food insecurity among adults with obesity: a quantitative and qualitative systematic review
Source: J Public Health (Oxf). 2024 Feb 26;46(2):230–49. doi: 10.1093/pubmed/fdae016 (PMC11141780; doi:10.1093/pubmed/fdae016)
Supplement: appendix3qualitativequalityappraisal_fdae016 [file appendix3qualitativequalityappraisal_fdae016.docx]

**Supplementary Table 3:** Quality appraisal of qualitative studies.

| **Author, Date** | **Aim?** | **Type of qualitative research?** | **3i) Setting?** | **3ii) participant selection?** | **Researchers' perspective?** | **5) Data collection?** | **6) Data analysis method?** | **QC methods?** | **7) Credible and clinically important?** | **8)Conclusions credible*?** | **9) Transferable findings?** |
| --- | --- | --- | --- | --- | --- | --- | --- | --- | --- | --- | --- |
| Cheung *et al*., 2015 | Understand the experience of FI and why some with FI do not experience obesity | Focus groups | Not stated | Randomised from patient list. No further explanation. | Over time the relationship of FI and obesity is not clear. Themes from focus groups were framed around the ecological model of health. | Focus groups were audio recorded, and FG guide was piloted before use. | Focus group themes were identified independently by reviewers, then a consensus was made regarding themes. | No software was used, nor was content saturation reached due to low participation or Cohen's Kappa calculated. | Yes | Only a few quotes were given though there were 21 participants. Potentially, outcome reporting bias could be at play.  Four focus groups were supposed to take place, but only one did. This is a low number considering it was a four-year study aiming to report the long-term effects of FI and BMI. | Potentially, as small sample size and no demographic data given about focus group |
| Cooksey Stowers *et al.,* 2020 | Understand FI and obesity from stakeholders' perspectives | Interviews | Via telephone for convenience and geographical reasons | Purposive sampling | Researchers utilised the equity-oriented systems approach, to understand if systemic inequality and historical oppression was at the route of FI and obesity | Interviews were semi-structured, translated verbatim. | Interviews were coded in NVivo V11 and analysed using thematic analysis. | Participants were recruited until content saturation was achieved and Cohen's Kappa = 0.81. | Yes | Yes, many quotes from interviews were used. | Yes, transferable to other US food banks and FI |
| Dressler & Smith, 2013 | Understand the experiences of women on SNAP by BMI group to assess differences in behaviour | Focus groups | Not stated | Participants were recruited from: food pantries, soup kitchens, homeless shelters, neighbourhood centres, libraries, the Special Supplemental Nutrition Program for Women, Infants, and Children, and by word of mouth. | Researchers utilised social cognitive theory framework for the focus groups. | Focus groups were audio recorded, transcribed verbatim. | Focus groups were transcribed independently and open coded. Themes were identified by researchers in a non-independent manner | Transcripts were coded until content saturation occurred. | Yes | Yes, many quotes from focus groups were used. | Yes, transferable to other countries where food assistant programs are used. |
| Kinsey *et al*., 2019 | Understanding the experience of SNAP participants and chronic illness on FI | three interviews with each participant over one month | At a children's hospital, participants homes, grocery stores, food pantries and soup kitchens. No explanation given for why the various locations were used. | A part of a larger trial, with purposeful sampling used to gain a more representative sample | Researchers highlighted the difficulties of managing chronic illness on SNAP | Semi-structured interviews using a piloted guide. | Interviews were analysed using Stata V14.2 and NVivo V11. Line-by-line reading allowed themes to be identified between two researchers. | Non stated, but consistency between the two researchers was 93% and they utilised the Consolidated Criteria for Reporting Qualitative Research guidelines. | Yes | Yes, many quotes from interviews were used. | Limited to other urban SNAP populations in USA |
| Knippen *et al*., 2020 | Understanding how eating behaviour is affected in older people in a community. | Focus groups and survey | Setting was not stated clearly | Recruitment flyers of people who took part in conjugate meals | Older participants have specific motivations regarding eating due to their psycho/social situations. Researchers split these views by two different generations. | Focus groups were audio recorded. However, due to participants speaking over one another they did not record demographics (FI and obesity levels) of who was speaking. | Researchers independently conducted line-by-line inductive coding to create themes. NVivo V12 was used for thematic analysis. | Did not state any QC method, such as Cohen’s Kappa. | Yes, with noted limitations | Yes, many quotes from focus groups were used. | Limited, as there was a very specific sample population. |
| Martinez-Jaikel & Frongillo, 2016 | Understand why and how women in Costa Rica are affected by food insecurity and excess body weight coexist | Focus group and in-depth interviews | Setting was not stated clearly | Participants were recruited from: Women’s Office–Municipality of Alajuela Database of women interested in participating in “healthy lifestyles groups.” | There is a link between FI and obesity | Focus groups and in-depth interviews, with the number of participants determined by data saturation. These were tape-recorded, transcribed verbatim, and then edited for accuracy. | ATLAS/ti 6.2, open coding, substantive codes and axial coding was used.  Not statistical but, to ensure data quality, peer debriefing through regular meetings of the primary researcher with other members of the research team enabled group discussion of emergent themes | Data was collected until content saturation occurred. | Yes | Yes, many quotes from interviews were used. | Transferable to other low/middle income urban societies. |
| Myers *et al*., 2020 | Understand FI obesity experience | Interview | Setting was not stated clearly | Convenience sampling from participants who consented previously. No more data given. | Hypothesis about psychological impact is the basis of questions | In-depth semi-structured interview were undertaken in-person, audio recorded and recorded verbatim. | MAXQDA software was used to analyse data via thematic analysis. | Data was collected until content saturation occurred. | Yes | Yes, many quotes from focus groups were used. | Transferable to others with same demographics, though a small sample size was used. |
| O'Malley *et al.,* 2012 | Understanding the viewpoint of a family who are FI and obese | Case study/commentary | Not stated | N/A | Commentary on specific situation | N/A | None | None | Limited clinical importance | Limited due to case study design | Limited transferable findings |
| Papan & Clow, 2015 | Understand how women in low-income areas experience of inequalities, obesity and FI | Focus groups | Setting not clear, from information available likely to be community centres | Recruitment aided by community centres and local organisations, with wide ethnic diversity. No other information available | Researchers based focus groups on feminist, Indigenous and participatory methodological frameworks | Unclear. No mention of audio recording, translating, or how themes were uncovered. | None stated | None stated | Yes | Yes, many quotes from focus groups were used, but limited explanation of focus group methodology. | Transferable to other areas in Canada or low-income women. |
| Taylor *et al*., 2019 | Understand the relationship between FI and weight loss in parents | Interviews | Public libraries, churches, or participants’ homes depending on participant preference. | Recruited from sites that serve as proxies for low income | Society is pressuring Western's to be thin, but this has not been explored in the context of eating behaviours for those who are FI | Interviews were semi-structured in-depth interviews based on USDA 18 item food security scale. Interviews were videotaped and transcribed verbatim. | Interviews were coded using a basic inductive approach, with NVivo 11. Coding was completed by one researcher and reviewed by another. | None stated. | Yes | Yes, many quotes from interviews were used. | Yes, to other high-income countries |
| Taylor *et al*., 2021 | Understand the relationship between mental health and FI | Interviewed individually | Setting was not stated clearly | Participants were recruited from various sites in Connecticut, USA that serve as proxies for income, such as Head Start and food pantries. | Researchers suggest is a link between mental health and FI. | Interviews were semi-structured, with scripted and unscripted probes, and was audio recorded. Interviews were transcribed verbatim. Limited further detail. | Interviews were coded using a basic inductive approach, with NVivo 11. Coding was completed by one researcher and reviewed by another. | Non stated. | Yes | Yes, many quotes from interviews were used. | Yes, to other high-income countries |
| Thompson, 2018 | Understand perspectives of stakeholders and clients of the UK food banking system | Interviews | Various locations, including food banks, cafés and supermarkets | staff recruited during ethnographic observations via snowball sampling and those recruited then approached clients. | Researchers used critical ground theory (CGT) and participatory philosophy with inherent social action agendas | Interviews were transcribed verbatim. | Data was analysed using CGT, with NVivo 10. | Not stated. Researchers state that the use of CGT means that data collection will never be saturated. | Yes | Yes, many quotes from interviews were used. | To other UK food banks and high-income countries. |
| Wiig & Smith, 2008 | Understanding shopping choices of those who use food stamps | Focus groups | Not stated | no info on recruitment other than flyers | Education on nutrition considered by authors to be important. Focus groups were framed around social cognitive theory. | Focus groups were audio taped, transcribed verbatim. | Researchers analysed independently via open-coding methods, to create themes. | Non stated. | Yes | Yes, many quotes from interviews were used. | Transferable to USA and other countries of similar demographics due to large sample size |

Qualitative Analysis Appraisal. Based on Greenhalgh (2019) principals for quality appraisal. BMI = Body mass index; FI = Food insecurity; QC = quality control; SNAP = Supplemental Nutrition Assistance Program; USDA = United Stated Department of Agriculture.

* = Conclusions have been summarised in Table 2 of the main text.
